# Supplementary figures and images for: A composite subunit vaccine confers full protection against Buruli ulcer disease in the mouse footpad model of Mycobacterium ulcerans infection
Source: PLoS Negl Trop Dis. 2025 Feb 21;19(2):e0012710. doi: 10.1371/journal.pntd.0012710 (PMC11918321; doi:10.1371/journal.pntd.0012710)

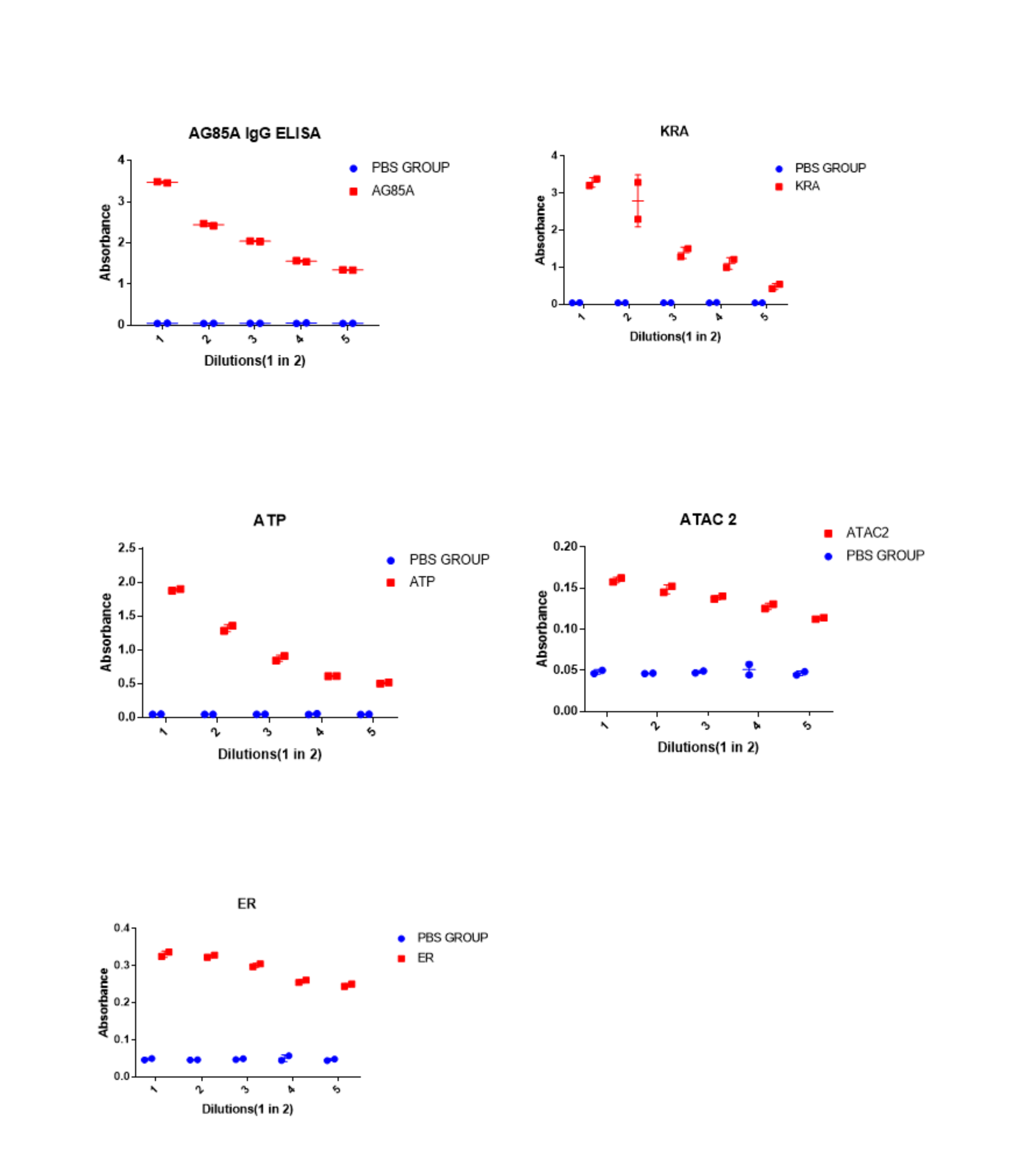

Supplement: S1 Fig — Shown are two-fold dilutions of sera starting from 1:100, in comparison to PBS immunised mice. The best titrating responses was in the Ag85A and KRA vaccinated groups while ATAC2 gave the poorest response. N = 2. (TIFF) [file pntd.0012710.s001.tiff]

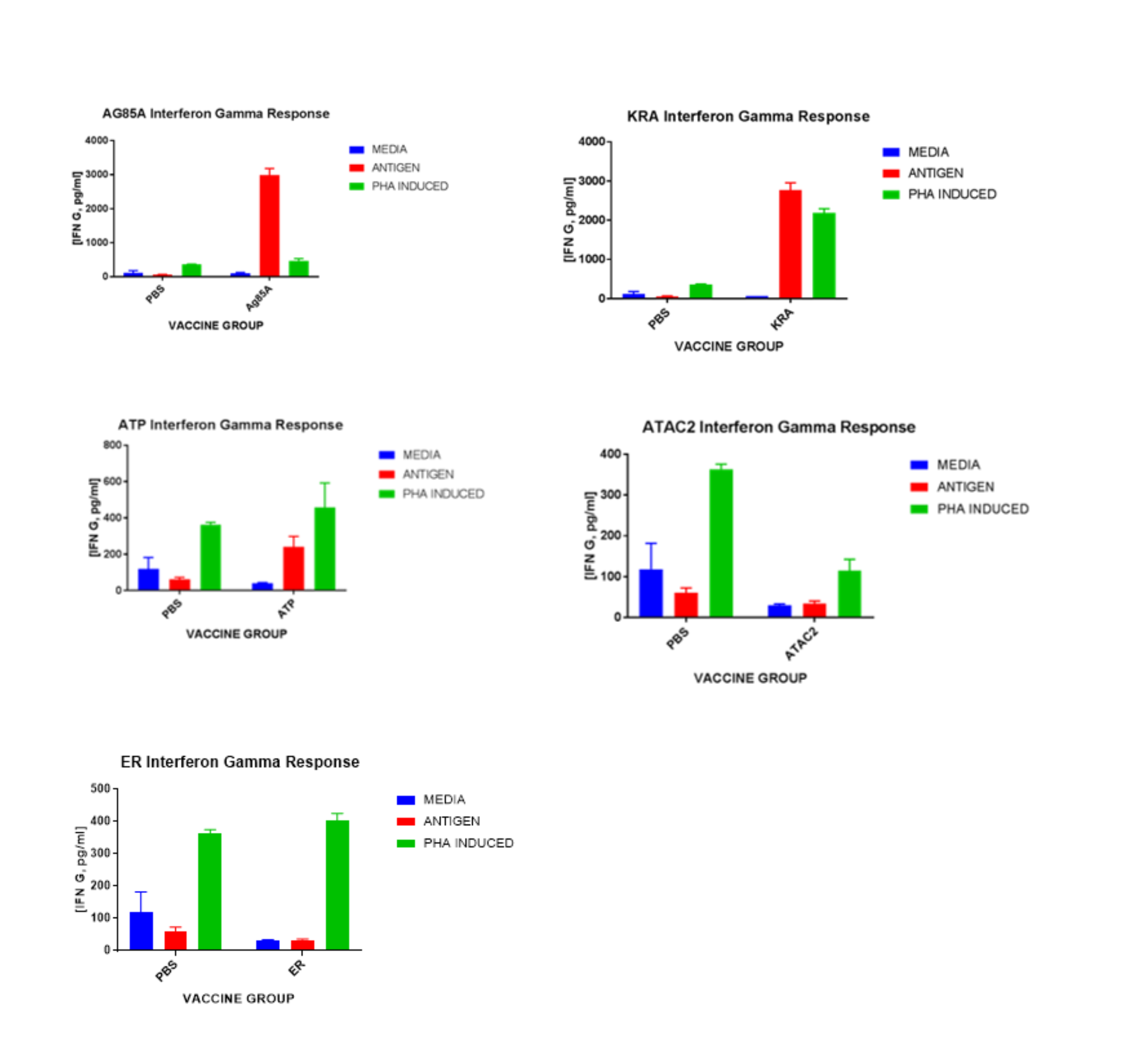

Supplement: S2 Fig — The colours indicate what the recall material was.i.e., Blank media (Negative control), Antigen of interest/immunisation and the Phytohemagglutinin (PHA) which was used as a positive control. Error bars represent standard deviation of the mean and bars are means of triplicate wells. N = 2. (TIFF) [file pntd.0012710.s002.tiff]

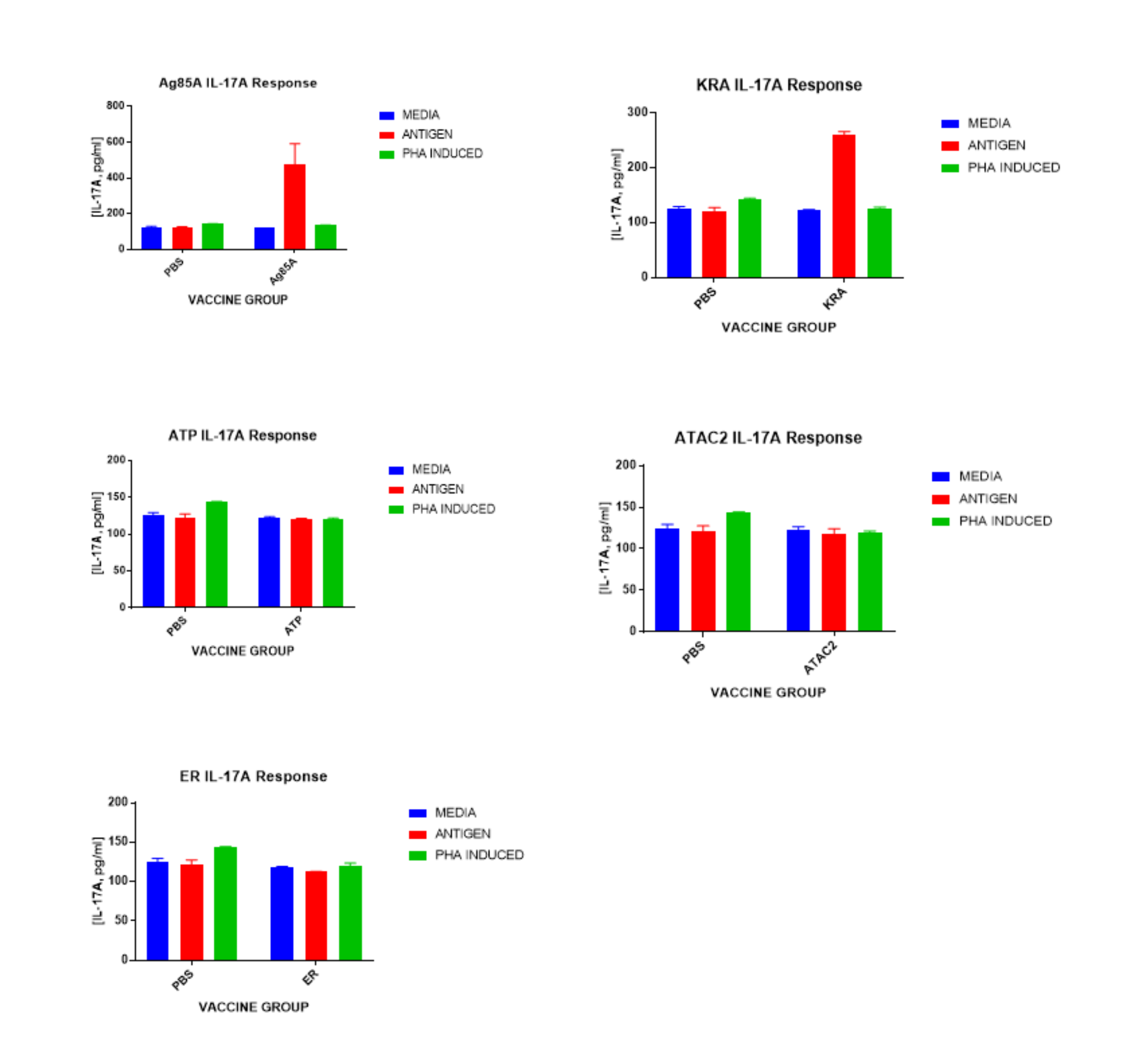

Supplement: S3 Fig — The colours indicate what the recall material was.i.e., Blank media (Negative control), Antigen of interest/immunisation and the phytohemagglutinin (PHA) which was used as a positive control. Error bars represent standard deviation of the mean and bars are means of triplicate wells. N = 2. (TIFF) [file pntd.0012710.s003.tiff]

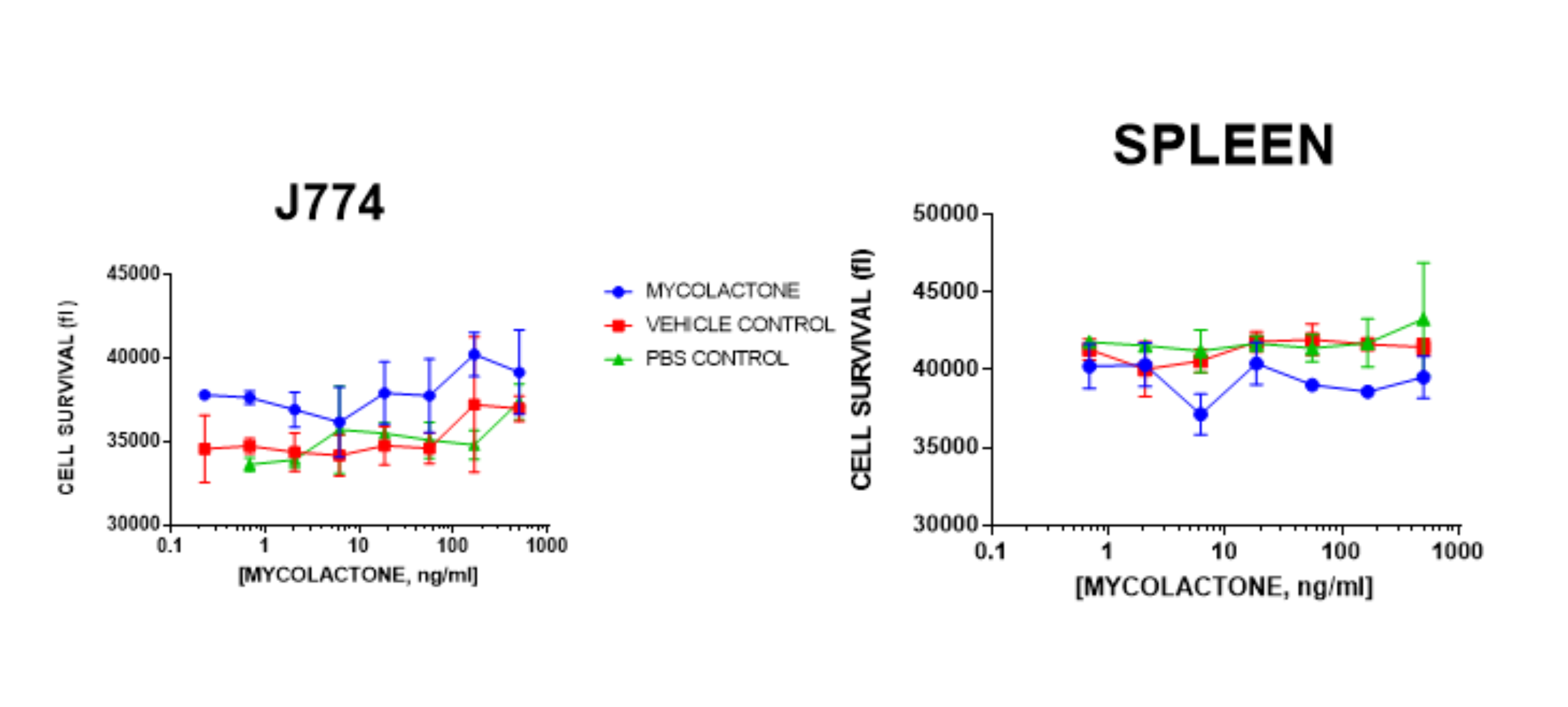

Supplement: S4 Fig — Cells after 72 h of incubation with different concentrations of mycolactone were incubated with Resazurin for 4–6 hours to determine survival. There is a direct relation between cell viability and absorbance (Excitation wavelength of 540 nm and Emission wavelength of 580 nm). J774 and mouse spleen cells maintained high survival rates. Error bars represent Standard Deviation from triplicate measurements. (TIFF) [file pntd.0012710.s004.tiff]

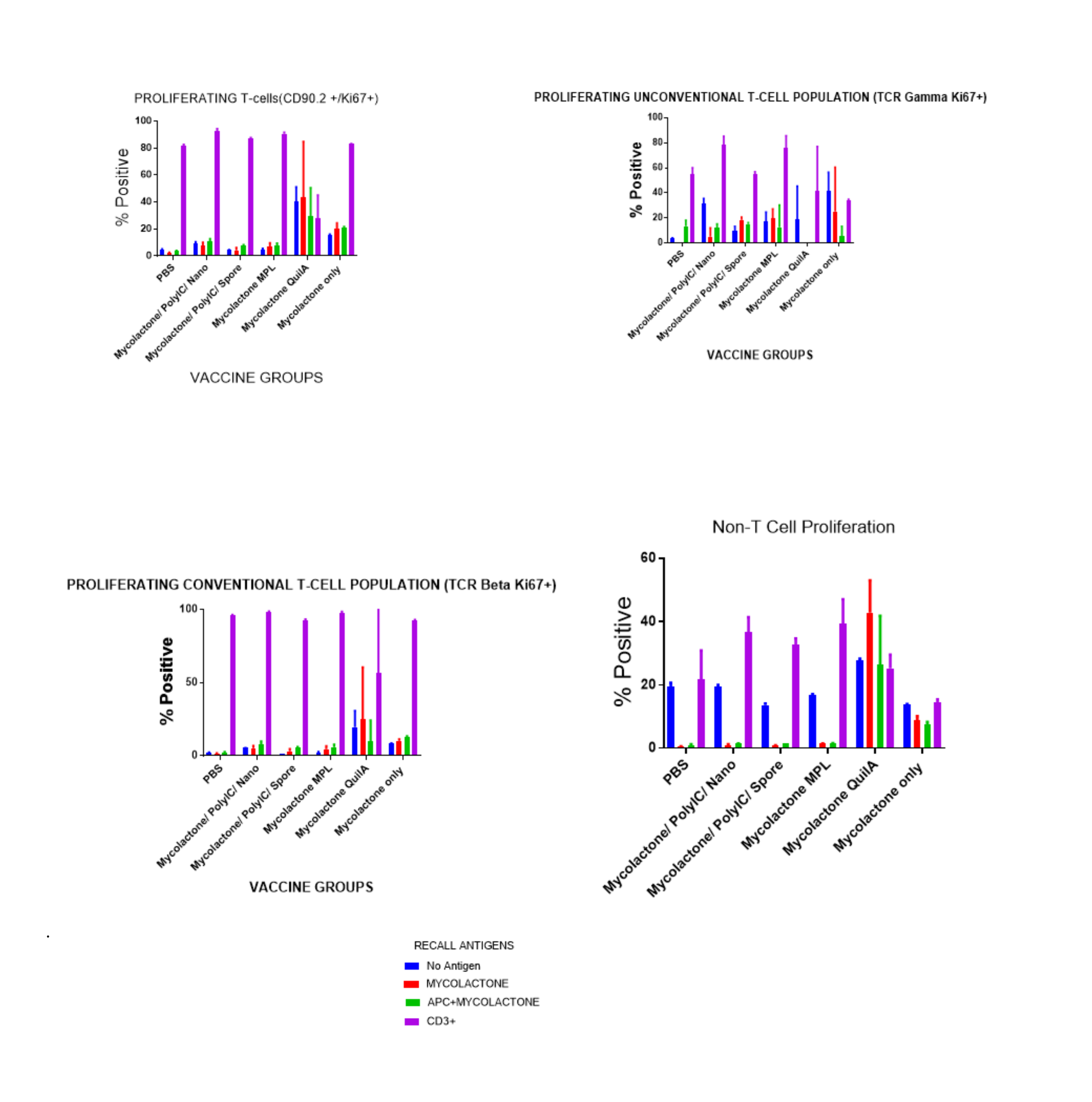

Supplement: S5 Fig — Bars are colour-coded by recall antigen. APC stands for antigen-presenting cells; these were enriched by splenocyte adherence to culture flask, pretreated with mycolactone and then combined with whole splenocytes). Anti-CD3 antibody was used as a positive control for T cell proliferation. Efluor 780 was used to monitor cell viability, while-Ki67 was used as a marker for proliferation. Proliferating cells are expressed as percentage of total cells. Error bars indicate Standard Deviation from triplicate measurements. Combined splenocytes from 2 mice. (TIFF) [file pntd.0012710.s005.tiff]
