# Supplementary material for: A composite subunit vaccine confers full protection against Buruli ulcer disease in the mouse footpad model of Mycobacterium ulcerans infection
Source: PLoS Negl Trop Dis. 2025 Feb 21;19(2):e0012710. doi: 10.1371/journal.pntd.0012710 (PMC11918321; doi:10.1371/journal.pntd.0012710)
Supplement: Attachment — Submitted filename: Reply to the Reviewers - BU paper.docx [file pntd.0012710.s007.docx]

**Reply to the Reviewers**

We thank all three reviewers on critical assessment of our manuscript and their comments. We have prepared a revised version of the manuscript in which new paragraphs and sections were added in response to the Reviewer’s requests, and they are marked in red. We have also included five supplementary figures to provide further evidence, as requested by the Reviewers.

Thus, we have addressed specific points raised as follows:

**Reviewer 1**

Methodology: we have provided further methodological detail which can be seen marked in red in the revised version

Potential challenges for translation: We have added a new section at the end of Discussion, addressing what we think might be the main challenges in translating our research into clinical testing and development.

**Reviewer 2**

We appreciate the Reviewer’s concerns and have now provide further evidence and explanations, where possible. In some instances, however, we do not agree with the Reviewer and explain why.

1. **Using mycolactone (ML) in native form**: Unlike other toxins which are proteins and typically multi-subunits that can be attenuated by removal of the toxic component (cholera, diphtheria, botulinum etc), ML is a macrolide and attenuation requires chemical synthesis of the modified molecule, which is beyond our expertise. However, the main reason we included native ML in our vaccine formulation is that, as we say it in the manuscript ‘to calibrate the immune responses to selected antigens, so that they mimic those induced during natural infection’. Because, using antigens alone in the past studies has not been successful, we needed a new approach, and with this new approach we achieved complete protection. Would ML be safe to apply in humans? It is possible, because it would be a controlled single low dose rather than continuous production as is the case during infection. However, if attenuated form of ML were to become available and can reproduce Burulivac vaccine efficacy, then we agree that that would be a safer option.

To this effect and in response to this comment from the reviewer, we added a paragraph in the Discussion that includes: ‘We argue though that at a fixed low dose as applied in this study, and in absence of live *Mu* that would continue to generate it, ML is very unlikely to cause localised tissue pathology. Indeed, in our mouse experiments we did not observe one, in any of mice inoculated with the vaccine dose of ML. Progressive swelling was observed only in animals that were pathogenically challenged with live *Mu*. Also, although ML alone induced local tissue apoptosis in the guinea pig skin model, this required a dose of 0.1 mg (8), which is 200-fold higher than what we used in our vaccine study in mice. It is therefore reasonable to argue that there is a window of immunomodulatory effect without significant cytotoxicity, but exploring this in detail was beyond the scope of the current study.’

2. **ML as immunomodulator vs target for immune system**: Immunomodulatory properties of ML are widely reported in BU literature, and we cite some of it in the Introduction (Refs 10 and 11). Wether ML is target for the immune cells is much less evidenced. We did generate some evidence for that during this project, which is now included as supplementary figures S4 and S5, but further work is required to delve deeper into it before we could propose a mechanism. Therefore, inclusion of ML in our vaccine formulation is based on both these properties of ML, but we agree with the Reviewer that further work is required, and we state so in the Discussion.

With regards to antibody response to ML, we could not detect them in sera using the conventional ELISA approach. The Reviewer states that ‘other research groups have reported the development and use of ELISA’ but we could not a reliable protocol, despite contacting several BU groups. It is true that some literature exists (for example, [10.1126/sciadv.aax7781](https://doi.org/10.1126/sciadv.aax7781)) reporting on antibodies to ML in skin lesions (but not sera), but upon close scrutiny, the data do not seem very robust and the ELISA protocol that includes steps such as ‘coated plates were stored at −20°C in the dark for several weeks’, does not lend itself well to reproducibility. Likewise, monoclonal antibodies to ML have been made by one research group ([10.1371/journal.pntd.0004808](https://doi.org/10.1371/journal.pntd.0004808)) but that required conjugation of ML to a carrier protein, and is not a protocol for detection of vaccine induced antibodies.

3. **The rationale for the choice of Ag85A and KRA antigens**: we fully agree with the Reviewer that we needed to provide some rationale. In fact, during the project we produced several antigens and preliminarily tested their immunogenicity in mice. Choice of Ag85A and KRA was based on their strongest immunogenicity (antibody and cellular responses) and we now provide evidence for this in the supplementary figures S1, S2 and S3, and address this in the main text of the manuscript.

As for dose finding experiments, we agree that this would be important to do. We could not do it because of the composite nature of the vaccine formulations which would require many experiments to do it for all 3 antigens and combinations. We state this in the Discussion when addressing the shortcomings of our study and the possibility of redundancy. However, as our vaccine formulation conferred absolute protection in the mouse footpad model, we are unlikely to be far off from optimal antigen doses.

4. **Comparison of vaccine formulations in Arm A ‘impossible’**. We respectfully disagree with the reviewer, as all formulations and/or regimens in Arm A reflect what would be the final vaccination scenario in humans, and their head-to-head comparison is fully valid. For example, BCG or MuΔ are live attenuated organisms and are given only as a single dose. Subunit vaccines can be given as a single dose, or boost doses, whether homologously (i.e. Burulivac followed by Burulivac) or heterologously (i.e. live vaccine as prime, followed by Burulivac as boost). Therefore, the priority here was to identify the best vaccine regimen against infection that could be translated into human application, and we did so, identifying those that are fully protective.

The Reviewer makes a valid point about the Arm B experiment, where individual Burulivac components were only tested as boost to MuΔ and not without it. That is due to logistical limitations and having multiple components and regimens. These are very complex and expensive experiments, and some compromises had to be made. Even so, the Arm B shows that boosting MuΔ with individual antigens was inferior to boosting with complete Burulivac formulation which had all three antigens. However, we were careful to state ‘A weakness of our study is that due to logistical constraints, we could not test all possible antigenic combinations or vaccine regimens to ensure that there is no redundancy. This remains to be established in future studies.’

**Reviewer 3**

We thank the Reviewer for positive evaluation of our work and understand that they had no concerns to raise.
